# CKD as an independent driver of polypharmacy

**DOI:** 10.1093/ckj/sfag184

**Published:** 2026-06-03

**Authors:** Rafael Santamaria, Carlos Escobar, Ignacio Hernández, Beatriz Palacios, Unai Aranda, Roberto Alcázar

**Affiliations:** Nephrology Service, Reina Sofia University Hospital, Maimonides Institute for Research in Biomedicine of Cordoba (IMIBIC), University of Cordoba, Cordoba, Spain; Cardiology Service, University Hospital La Paz, Madrid, Spain; Atrys Health, Madrid, Spain; BioPharmaceuticals Medical, AstraZeneca, Madrid, Spain; Global Medical Affairs, BioPharmaceuticals Medical, AstraZeneca, Gaithersburg, MD, USA; Nephrology Service, University Hospital Infanta Leonor, Madrid, Spain

To the Editor,

A clinically important yet underexplored question is whether chronic kidney disease (CKD) contributes to medication burden beyond the effect attributable to chronological age alone. In this retrospective observational study of 70 385 adults, medication burden increased with age, worsening kidney function and higher albuminuria. Strikingly, younger adults with impaired kidney function displayed medication profiles comparable to those of older adults with preserved kidney function. This finding is consistent with a shift in polypharmacy patterns and premature pharmacological ageing. Therefore, CKD should therefore be recognized as an age-independent driver of treatment complexity, calling for stage- and age-adapted medication stewardship.

CKD is a major global health challenge that requires complex medical management, often involving multiple medications [1, 2]. Polypharmacy, defined as the concurrent use of ≥5 drugs is highly prevalent in CKD, especially in advanced stages [3]. While necessary for treatment, it is associated with a higher risk of adverse drug reactions, drug–drug interactions, and poor adherence [1, 2]. Older adults with CKD face greater medication burdens due to comorbidities such as diabetes and heart disease [4, 5].

However, an important and still insufficiently explored question is whether CKD contributes to medication burden beyond chronological age alone. To address this gap, we used real-world clinical data to examine how medication burden varies across CKD stages and age groups.

We conducted an observational, retrospective study using the BIG-PAC database including 70 385 adults with ≥1 measurement of estimated glomerular filtration rate (eGFR) and albuminuria close to January 2018 and separated by a maximum of 3 months, of whom 30.0% had CKD [4]. Polypharmacy increased with advancing age, declining kidney function, and higher levels of albuminuria.

**Figure 1:**
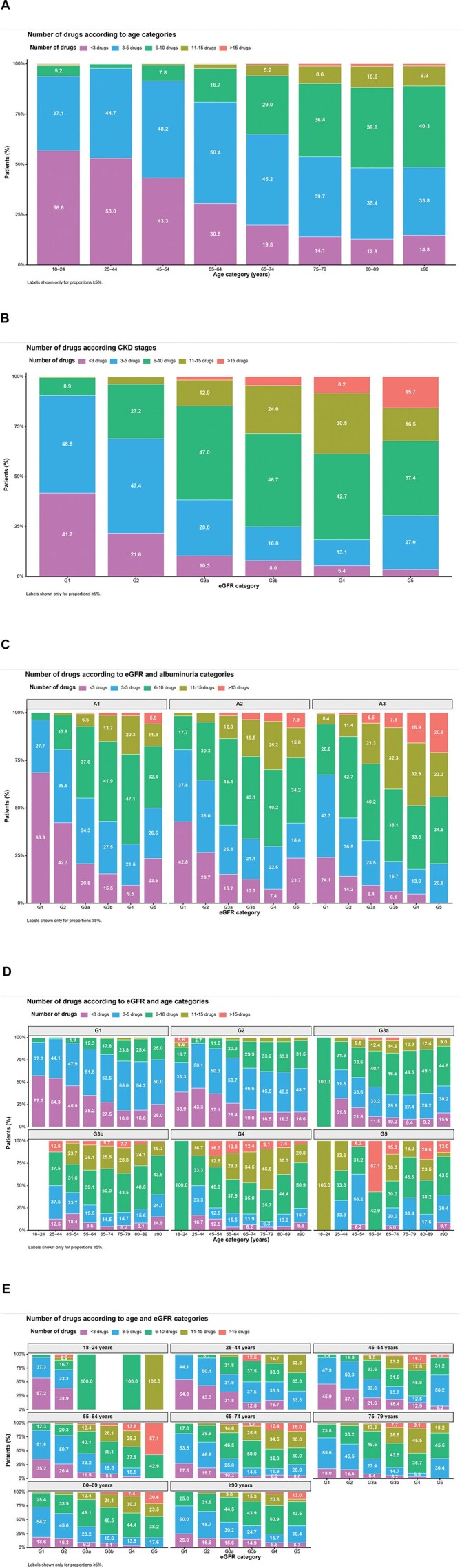
Number of drugs according to age categories (A), CKD stages (B), eGFR and albuminuria categories (C), and eGFR and age categories (D and E).

Medication use increased steadily with age, with most younger adults (18–44 years) taking fewer than three drugs. The proportion of individuals using 3–5 medications peaked in midlife (45–64 years), whereas from age 65 onward, a higher medication burden became increasingly common. Use of 11–15 medications was rare in younger adults but increased to 10.6% in adults aged 80–89, with some individuals exceeding 15 drugs (Fig. 1A).

The number of prescribed drugs increased as kidney function declined. In G1–G2, most patients took fewer than five medications. From G3a onwards, the proportion of patients receiving ≥6 drugs rose sharply (Fig. 1B). More importantly, at any given eGFR category, the presence of albuminuria consistently moved prescribing patterns towards a greater degree of polypharmacy (Fig. 1C).

Finally, we found that medication burden was jointly shaped by age and kidney function (Fig. 1D and E). Within each age group, declining eGFR was consistently associated with a shift towards greater polypharmacy. This effect was particularly evident in younger adults, in whom reduced kidney function markedly shifted prescribing patterns towards profiles more typically seen at older ages. Conversely, for a given CKD stage, older age was also associated with greater polypharmacy, although this age-related gradient became progressively less evident in advanced CKD (from G3b onwards).

Recent data show that >80% of CKD patients experience polypharmacy, particularly in advanced stages, which is associated with adverse clinical outcome [1]. Similarly, polypharmacy is known to be more prevalent in older adults, largely reflecting the greater burden of comorbidity [5]. Our findings add an important nuance to this framework. Although medication burden increased with both age and worsening kidney disease, the effect of CKD appeared to extend beyond chronological ageing alone. Within each age stratum, declining eGFR was associated with a clear shift towards greater polypharmacy, an effect that was especially marked in younger adults. Indeed, younger patients with impaired kidney function often displayed medication profiles comparable to those of older individuals with better preserved kidney function, consistent with the concept of premature pharmacological ageing. Furthermore, the presence of albuminuria shifted the distribution of drug use towards greater polypharmacy, suggesting that medication burden in CKD is driven not only by reduced kidney function but also by the broader clinical complexity captured by albuminuria.

In this context, it is essential to implement strategies to address inappropriate polypharmacy and promote deprescribing, thereby minimizing adverse outcomes through a comprehensive medication management process [2]. Individualized interventions are needed across the CKD spectrum. For younger patients, focus on prevention and lifestyle changes to slow CKD progression. In older patients, structured medication review and deprescribing strategies should be emphasized to reduce adverse drug events and improve adherence [2, 5]. Albuminuria significantly influences polypharmacy, particularly in advanced CKD, so early preventive measures are essential to reduce polypharmacy risk [2, 4]. Overall, these findings highlight the need for a multidisciplinary approach to CKD care, especially in later stages, where coordinated efforts among nephrologists, pharmacists, and primary care providers are necessary to optimize medication management [2].

In conclusion, our real-world study shows that polypharmacy in CKD reflects the combined influence of age, reduced kidney function, and albuminuria, but also suggest that CKD shifts medication burden beyond that expected for age alone. Recognizing CKD as an age-independent driver of treatment complexity may help refine risk stratification and support earlier, more individualized medication stewardship across the CKD continuum.
